# Editorial: Country profile of the epidemiology and clinical management of early childhood caries, volume III

**DOI:** 10.3389/froh.2024.1373452

**Published:** 2024-03-12

**Authors:** Moréniké Oluwátóyìn Foláyan, Robert J. Schroth, Francisco Ramos-Gomez, Maha El Tantawi

**Affiliations:** ^1^ Early Childhood Caries Advocacy Group, University of Manitoba, Winnipeg, MB, Canada; ^2^ Oral Health Initiative, Nigerian Institute of Medical Research, Lagos, Nigeria; ^3^ Department of Child Dental Health, Obafemi Awolowo University, Ile-Ife, Nigeria; ^4^ Africa Oral Health Network, University of Alexandria, Alexandria, Egypt; ^5^ Dr. Gerald Niznick College of Dentistry, University of Manitoba, Winnipeg, MB, Canada; ^6^ Division of Preventive and Restorative Oral Health Sciences, UCLA School of Dentistry, Los Angeles, CA, United States; ^7^ Department of Pediatric Dentistry and Dental Public Health, Faculty of Dentistry, Alexandria University, Alexandria, Egypt

**Keywords:** sustainable development goals, oral health, disease burden, technology, sugar intake

**Editorial on the Research Topic** Country profile of the epidemiology and clinical management of early childhood caries, volume III

Early childhood oral health is a critical aspect of overall well-being, and recent research has shed light on various dimensions of this complex issue. A comprehensive examination of eight manuscripts provides a nuanced understanding of the challenges and opportunities in managing early childhood caries (ECC) and fostering optimum oral health practices among children. From dentist perspectives to interventions in low socioeconomic settings, complex case reports and innovative online platforms, these studies offer valuable diverse insights that collectively contribute to our broader understanding of pediatric oral health and how to navigate the landscape of ECC management.

The high prevalence of ECC in Myanmar (1) is an example of the high burden of this oral disease experienced by many children globally, especially those in low- and middle-income countries. Children from low socioeconomic households, regardless of country or ethnicity, are at higher risk for ECC. One contributing factor is the gap in oral health knowledge and practices among parents and caregivers. Haque et al. showed that despite positive maternal attitudes about preventive oral health measures, the awareness about the need for regular dental examinations is poor. In addition, maternal smoking, and delayed initiation of oral hygiene practices among preschool children increases the risk for ECC (Sobiech et al.).

The multifactorial risk nature of ECC presents inherent challenges to its prevention and management. The case report by Fan et al. recommends that multidisciplinary approaches are needed to ensure that children affected by ECC have access to oral health education, diet and nutrition information, and care, including restorative treatment. It is also important that dental providers embrace non-restorative management of caries, when possible. Few ECC management approaches have outlined the importance of sustaining follow-up care as highlighted by Fan et al. Further studies are needed to identify suitable and culturally appropriate models to support families with children at risk for ECC to ensure continuing preventive and curative care. Abreu-Placeres et al. also identifies the need for effective prevention and management strategies, including maintaining oral health practices and controlling sugar consumption.

To curb the burden of ECC, several key steps need to be taken. First, concerted efforts are needed to improve access of young children to oral care, beginning with the first dental visit at or before the first birthday. Access can also be improved by addressing financial barriers to oral health care. Canada, like a few other countries, has improved the access of children to oral health care through the launch of the Canadian Dental Care Plan in 2024 (2, Schroth et al.). More countries need to improve access of children to preventive and curative ECC care. Access to oral healthcare can also be improved by ensuring dental professionals are comfortable and equipped to care for young children. Levesque et al. identified gaps in dentist awareness about ECC, particularly among general dentists, emphasizing the need for targeted education and support, especially for male practitioners, to use evidence-based topical fluorides and sealants to prevent ECC. Schroth et al. also, highlighted that ECC prevention can be extended to non-dental primary healthcare settings by building the competency of diverse healthcare professionals to use context appropriate, time efficient, user-friendly caries risk assessment tools to screen, identify preschool children at high risk for ECC, and refer them to a dental provider promptly.

Furthermore, investing in building the competency of caregivers to institute measures to prevent ECC is critical as this further reduces the risk of caregivers being charged with child neglect (3, 4, Foláyan et al.). One possible way to engage parents is using technology and the social media. Online caries management platforms should be considered to promote early childhood oral health as this has been shown to be more effective in reducing sugar intake and increasing the tooth brushing time than traditional education platforms (Yan et al.).

The insights gained from the publications in this research topic contribute to a broader understanding of early childhood oral health, offering evidence to support new policies and interventions that align with the World Health Organization's Sustainable Development Goals (SDG). The studies clearly show that the strategic actions needed to improve the epidemiological profile and clinical management of ECC aligns with the SDG 3, 4, 9, 10 and 17. SDG 3 ensures healthy lives and promote the well-being of infants, toddlers and pre-school children; SDG 4 ensures inclusive and equitable quality education on the prevention of ECC for caregivers and general dentists; and SDG 9 refers to the use of innovative digital technologies to manage ECC. In addition, SDG 10 addresses inequalities, and SDG 17 highlights the importance of partnerships. Each of the eight studies can be linked to any of these SDGs thereby complementing ongoing research on the links between the SDGs and ECC (3–8).

In conclusion, this paper provides insight into why ECC may be considered a “wicked problem” that comes with lots of management challenges because it has multiple causes that intersects with many other problems (9). Because of this, ECC needs multiple solutions on many levels to address its management challenges. The eight studies in this Research Topic provide insights into the multifaceted risk factors and management modalities of ECC. Such a holistic overview deepens our nuanced understanding of pediatric oral health and offers actionable strategies for policies and interventions that fosters a collective stride towards achieving the SDGs.
